# *Mesona chinensis* polysaccharide accelerates the short-term retrogradation of debranched waxy corn starch

**DOI:** 10.1016/j.crfs.2022.09.018

**Published:** 2022-09-22

**Authors:** Wenhao Xiao, Jinwang Li, Mingyue Shen, Qiang Yu, Yi Chen, Jianhua Xie

**Affiliations:** a State Key Laboratory of Food Science and Technology, Nanchang University, Nanchang, 330047, China; b China-Canada Joint Lab of Food Science and Technology (Nanchang), Nanchang University, Nanchang, 330047, China

**Keywords:** Debranched waxy corn starch, *Mesona chinensis* Benth polysaccharide, Gel, Retrogradation

## Abstract

The effect of non-starch polysaccharides on the structural and functional properties of native starch have been extensively studied. However, the effect of non-starch polysaccharides on the structural characteristics of debranched starch, a kind of enzymatic modified starch, remains unclear. The aim of this study is to investigate the effects of *Mesona chinensis* polysaccharide (MP) on starch retrogradation and structural properties of debranched waxy corn starch (DWS). The results showed that only appropriate addition of MP (0.5 or 1%) can effectively promote the short-term retrogradation of DWS, while excessive MP (3 or 5%) had a negative effect. Gel hardness results revealed that the short-term retrogradation (24 h) of DWS could be divided into two phases. The retrogradation of DWS-MP gels mainly occurred at first stage (0–4 h), which was demonstrated by the rapid increase of gel hardness and relative crystallinity in this stage. In the second stage (4–24 h), DWS-MP gels were more likely to undergo the aggregation of starch granules as proved by SEM and particle size results. The degree of short-range ordered decreased during the total retrogradation stage. Overall, this work aims to provide an insight into the effect of non-starch polysaccharides on the short-term retrogradation of DWS.

## Highlights

• Only the appropriate addition of MP could accelerate the retrogradation of DWS.
• The short-term retrogradation of DWS could be divided into two stages.
• Gel hardness and relative crystallinity increased significantly in the first stage.
• The degree of short-range ordered reduced monotonically with retrogradation time.
• Starch particles mainly underwent aggregation in the second stage.

## Graphical abstract

**Figure:**
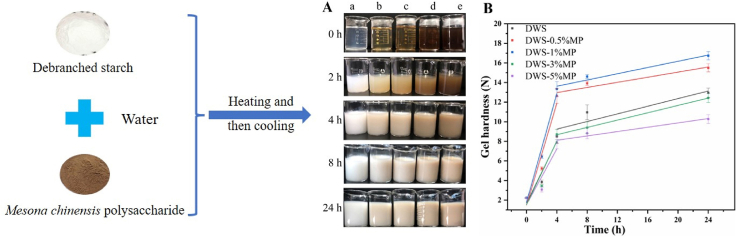

## 1 Introduction

In the process of hydrothermal gelatinization, the ordered structure, birefringent character and semi-crystalline structure of native starch are destroyed (Wang and Copeland, 2013), which is attributed to excess water entering the amorphous regions and then form hydrogen bonds with starch molecules, disordering the granule crystalline region of particle and resulting in the leaching of soluble polysaccharides (amylose) (BeMiller, 2011; Donmez et al., 2021). After cooling storage, starch molecules begin to reassemble into ordered crystalline structure due to water migration, and this process is called “starch retrogradation” (Luo et al., 2020; Wang et al., 2015). Especially stored at 4 °C, the retrogradation process of gelatinized starch is more easily to achieve. During such a process, amylose plays a regulatory role in a relatively short time.

In general, starch retrogradation is considered as an undesirable property because it reduces the starch quality, including decreasing nutritional quality, increasing hardness and shortening shelf life. However, everything has two sides. Starch retrogradation also has its commercial application values. For example, in the production of breakfast cereals and cooked rice, as retrogradation causes suitable hardening and reduces stickiness (Colonna et al., 1992; Karim et al., 2000; Chen et al., 2022). In addition, the preparation of resistant starch (RS) type III is mainly utilizing the retrogradation of starch. A typical method of this is debranched starch (DS). Starches have been debranched and recrystallized to prepare RS. DS is primarily composed of retrograded amylose because of its strong tendency to aggregate and reassociate. RS has been shown various physiological benefits in humans, including lowering the postprandial glycemic index, a prebiotic effect on colon flora, lowering blood cholesterol and preventing diabetes, and reducing the risk of colon cancer (Liu et al., 2020; Ozturk et al., 2009; Shi et al., 2013). DS is an enzymatic modified starch produced by enzymatic debranching, which results in linear short-chain amylose that can more readily rearranged into an ordered structure (Cai and Shi, 2014). Starch retrogradation is a complex process depending on various factors such as storage temperature and time, food composition, solid concentration and so on (Cai and Shi, 2014; Gonzalez-Soto et al., 2007; Lee et al., 2019; Ma et al., 2019; Morris, 1990). In recent years, the impact of different treatments on DS retrogradation and recrystallization, such as ultrasonication (Qin et al., 2020), annealing (Chang et al., 2021), heat-moisture (Chang et al., 2020), repeatedly crystallized (Zeng et al., 2015), and high-temperature pressure have been reported (Zhang et al., 2013). However, the effects of other components such as hydrocolloids on retrogradation of DS are not well documented.

According to the previous studies (Biliaderis et al., 1997; Ravindran and Matia-Merino, 2009; Yang et al., 2021), the addition of hydrocolloids can delay or facilitate the retrogradation of starch. *Mesona chinensis* polysaccharide (MP) has been proved that it has not only various biological activities, but also a significant impact on the textural, rheological, digestibility, microstructure and retrogradation properties of starch (Lin et al., 2017; Luo et al., 2020; Ren et al., 2020; Tang et al., 2017; Yuris et al., 2019). MP is an acidic anionic polysaccharide with a molecular weight of 1.4 × 10^^6^ Da. It mainly consisted of xylose and galacturonic acid in a molar ratio of 2.5:6.3 (Xiao et al., 2021). It was found that the addition of MP increased the tensile strength and elongation of sweet potato starch film (Ren, Wu, et al., 2021). Xie, Ren, Xiao, Luo, and Shen (2021) suggested that hydrogen bond played a dominant role in the textural properties of MP-tapioca starch gels. MP It was proved that MP not only could maintain the stability of maize starch gel, starch, but also could promote the retrogradation and the formation of ordered structure (Luo et al., 2020). Several studies have been carried out to investigate the effect of MP on the pasting and retrogradation properties of various natural starches. However, the effects of MP on retrogradation of DS are not well documented. Whether the addition of MP can also have a significant effect on the retrogradation properties of debranched waxy corn starch (DWS)?

Therefore, our first objective of this work was to study the effect of the time interval of retrogradation at 4 °C on rheology, gel, particle size, and short- or long-range ordered structure of DWS. The second objective was to find out if adding MP to DWS during cooling at 4 °C does accelerate its short-term retrogradation or not. This work will benefit to further understand and regulate the short-term retrogradation process of debranched starch.

## 2 Materials and methods

### 2.1 Materials

MP (30.6% netural sugar, 56.9% uronic acid, molecular weight was 1.45 × 10^6^ Da) was extracted from *Mesona chinensis* Benth by hot alkali extraction method (Lin et al., 2017). Waxy corn starch (98.7% amylopectin) was purchased from Shandong Huanong Special Corn Starch Development Co., Ltd. (Shandong, China). DWS was prepared according to our previous method (Xiao et al., 2022).

### 2.2 Preparation DWS-MP paste for retrogradation behavior analysis

DWS (5%, w/v) and different concentrations of MP (0, 0.5%, 1%, 3%, and 5%, w/w, based on starch dry weight) were mixed in a 50 mL EP tube. Then mixtures in boiling water were heated for 30 min to fully gelatinized. Then, the DWS-MP dispersion was cooled and then transfer into a 4 °C refrigerator for different storage time (0, 2, 4, 8, and 24 h). The retrograded DWS-MP gels were submitted to textural, rheological, and particle size distribution analysis, and then freeze-dried for further analyses.

### 2.3 Textural measurements

Gel textural properties were determined by a texture analyzer (TA-XT plus, Stable Micro Systems, UK) according to the method by Massarolo et al. (2019) with adaptations. The completely gelatinized DWS-MP suspension obtained from section 2.2 was transferred into a beaker and stored at 4 °C for various times (0, 2, 4, 8, and 24 h). Texture analysis of gels was performed using a P/0.5 R probe to puncture gels with 10 mm depth. The pre-test and post-test speeds were 2 mm/s, and test speed was 1 mm/s. The trigger force was 1 N and the trigger type was automatic (Xu et al., 2021).

### 2.4 Rheological measurement

Rheological characters of DWS-MP gels were conducted using a rheometer (DHR-2, TA Instruments Inc., USA) (40 mm diameter parallel plate, 0.5 mm gap) (Shi et al., 2021).

#### 2.4.1 Dynamic frequency test

The dynamic rheological properties (*G′*) were determined (0.5% strain, linear viscoelastic range). The frequency sweep data was fitted into the power law model:

G′ = *K* (ω) ^*z*^

where ω is the angular frequency, and the values of *z* and *K* reflect the type and strength of molecular interactions of the gels, respectively.

#### 2.4.2 Steady shear test

The steady shear flow tests were performed at shear rates ranging from 0.1 s^−1^–100 s^−1^ at 25 °C. The mechanical spectra were plotted in terms of apparent viscosity as a function of shear rate.

### 2.5 Particle size distribution

The particle size distributions of DWS-MP were determined by a laser diffraction instrument (Malvern Mastersizer 3000, UK) based on the method of Li et al. (2018). In brief, fresh starch gel was added to the sample cell (about 600 mL of distilled water) until the masking level reached 8%, and the rotational speed of agitator was set at 3800 rpm to prevent multiple scatter (ultrasound off). The refractive index of the dispersant was 1.33, and the refractive index of the particles was 1.47.

### 2.6 Fourier transform infrared spectroscopy (FT-IR)

FT-IR spectra and Deconvolved infrared spectra of the DWS-MP gels were obtained by using a FT-IR spectrophotometer (FT-IR, Nicolet 5700, USA) at room temperature. (Zhao et al., 2020).

### 2.7 X-ray diffraction (XRD)

X-ray diffraction patterns were performed by a Bruker X-ray diffractometer (D8 Advance; Bruker Inc., Germany).

### 2.8 Morphology

The morphological characterization of freeze-dried sample was observed with a scanning electron microscope (SEM) (JSM-6360LV, JEOL, Japan) (Cao et al., 2021).

### 2.9 Statistical analysis

Data were compared by using SPSS software (version 22.0, SPSS Inc., Chicago, IL, USA). All the figures were performed by Origin Pro (version 8.0) software (Stat-Ease Inc., Minneapolis, USA).

## 3 Results and discussion

### 3.1 Gel hardness

**Fig. 1:**
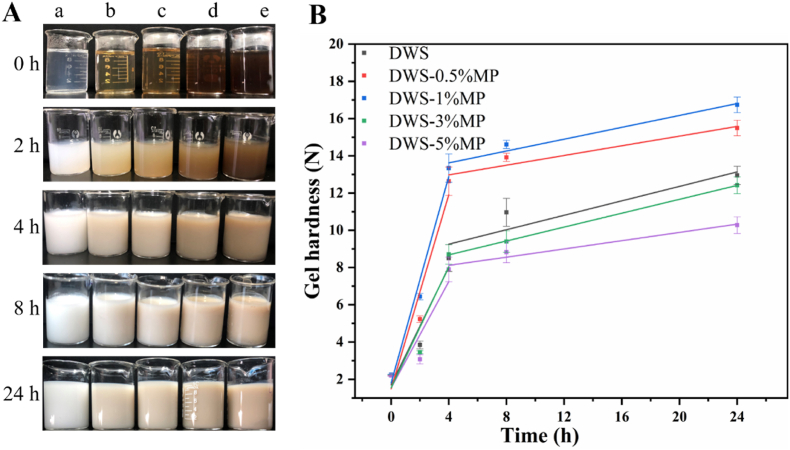
(A) Photographs from DWS gels with different concentrations of MCP (a: 0%, b: 0.5%, c: 1%, d: 3%, e: 5%) after being stored at 4 °C for different storage times. (B) First order linear fitting curve of gel hardness of DWS-MP gels at two stages (0–4 h and 4–24 h) during retrogradation process.

The visual appearance and gel hardness of DWS-MP gels are shown in Fig. 1. Gel hardness is highly related to retrogradation of starch (Du et al., 2019; Zhao et al., 2021). Without retrogradation at 4 °C, all DWS-MP gels were almost liquid (with very low hardness values) rather than dense gels (Fig. 1A). Therefore, rheometer and particle size analyzer failed to determine the data (lower than detection limit), so DWS-MP gels with 0 h retrogradation were not tested in later part. The hardness values of all DWS-MP gels increased with increasing storage time, which was attributed to the retrogradation and association of debranched starch to forms a gel network structure (Ding et al., 2020; Yu et al., 2009), resulting in reinforcement of the DWS gel. Another reason might be due to the interactions between DWS and MP (Ren et al., 2020). Compared with the DWS alone, the addition of MP caused a significant changed of gel hardness. As the amount of MP increased from 0 to 1%, the values of gel hardness increased from 12.95 to 16.74 N after 24 h storage, and subsequently decreased to 10.28 N when the amount of MP reached up to 5%. These results suggested that only the appropriate MP could improve the gel hardness of DWS gels, while excessive addition of MP had the opposite effect. Liu et al. (2018) also found similar results in wheat starch and MP system, which was attributed to high levels of MP forming a hydration film around of starch granule and retarding the agglomeration and rearrangement of starch molecules. During the short-term retrogradation process, DWS-1%MP exhibited higher gel hardness values than that of other groups. In addition, in order to reveal the relationship between the storage time and gel hardness of DWS-MP gel, first order linear fitting model was used to evaluate the rate of increase in gel hardness with storage time when the gels were stored at 4 °C for 24 h. There were two stages of gel strength enhancement with different rates during storage (Fig. 1B). 0–4 h was the first stage and 4–24 h was the second stage. In the first stage, the values of slope b_1_ increased first and then decreased with the addition of MP, suggesting that low levels of MP could accelerate the formation of gel network of debranched starch, while high level of MP hindered it. In the second stage, the values of slope b_2_ of all samples dropped dramatically (all b_2_ < 0.2) compared to the first stage b_1_, and there was no significant difference in b_2_ values between each group. According to the previous reports, the short-term retrogradation of starch was determined by amylose, which was a very fast process in short time (Gudmundsson, 1994; Xu et al., 2013). Therefore, in this study, it was reasonable to say that retrogradation of DWS mainly occurred in the first 4 h, and then the rate of retrogradation process decreased. By controlling the retrogradation time and retrogradation rate to alter textural parameters will make a lot of sense in food processing (see Table 1).

### 3.2 Rheology properties

**Fig. 2:**
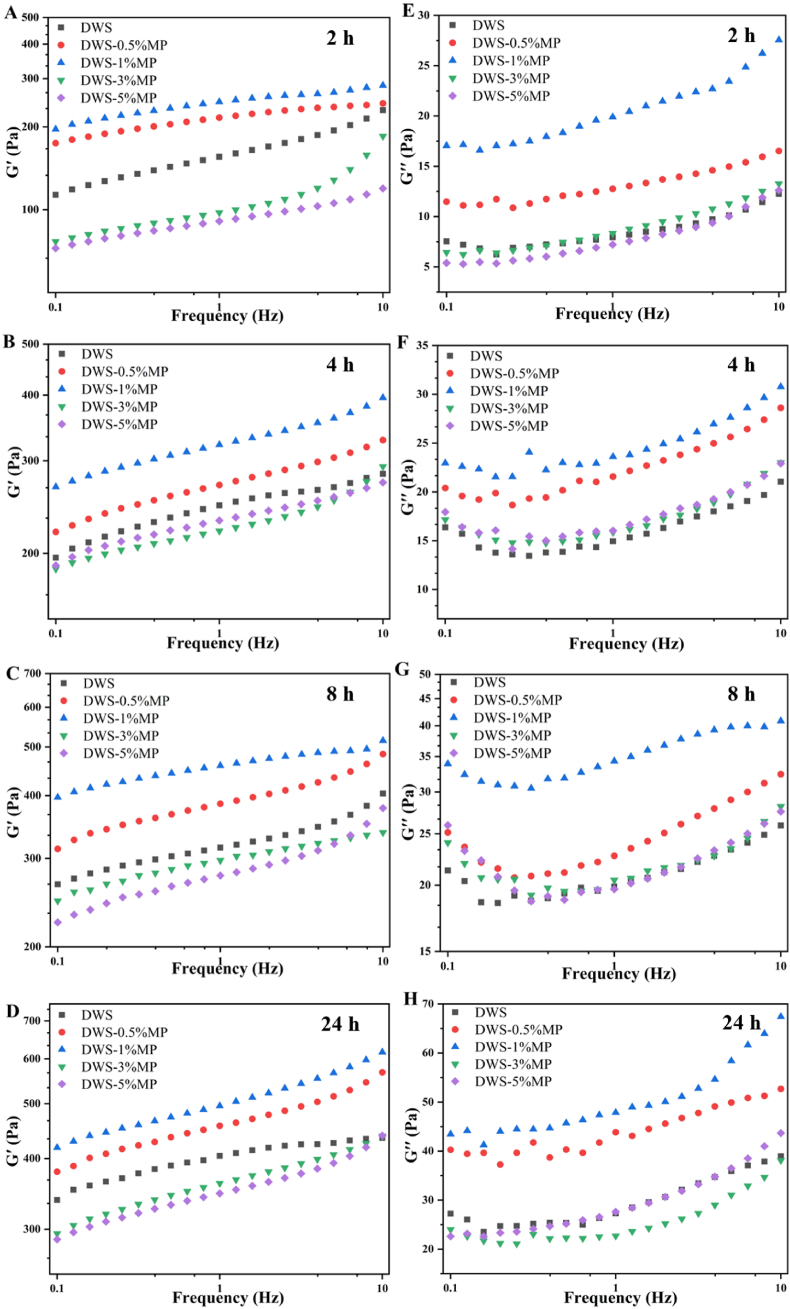
Dynamic rheological properties of DWS-MP gels at different storage times.

The dynamic modulus *G′* and *G′′* as a function of angular frequency of all DWS-MP gels during storage are presented in Fig. 2. As the angular frequency increased, the dynamic modulus of DWS-MP gels increased throughout the test frequency range, indicating that all DWS-MP gels exhibited a frequency-dependent behavior. Furthermore, *G′* of all samples was consistently higher than *G′′* at all the time, meaning that DWS-MP gels was a weak solid gel (Okonkwo et al., 2021). *G′* and *G′′* seemed to first increase and then decreased with the addition of MP (Fig. 2). The results were consistent with our previous study (Xiao et al., 2021). These results indicated that low concentrations MP enhanced the viscoelasticity while high levels of MP reduced the viscoelasticity of DWS gel. The enhancement of *G′* and *G′′* might be due to the fact that the entanglement nodes between the starch molecular chains increased in the mixture system (Liu et al., 2018; Takahashi and Fujita, 2017). The power law model fitting parameters (*K* and *z*) of *G′* are shown in Table 2 (R^2^ = 0.86–0.99). As it was evident, MP had a significant effect on the fitting parameters (*P < 0.05*). Coefficients *K* represents the magnitude of *G′* at a specific frequency. The value of *K* for DWS-1%MP was larger than those of the others, which meant that the incorporation of 1%MP resulted in the formation of stronger gel structure of DWS gels (Chen et al., 2021; Pourfarzad et al., 2021). This result could be proved by the results obtained from gel hardness values. It is well-known that the values of *z* are associated to the characteristics of elastic gels. Lower values of *z* represent more elastic behaviors in starch gels (Yousefi and Razavi, 2015). As it can be observed, there was no obvious trend of *z* value compared to the *K* value, and it might be due to the fact that the values of *z* were lower than 0.2 for all samples, which suggested that all DWS-MP gels exhibited an extremely strong elastic behavior. Thus, it was difficult to show significant changes in *z* value.

**Table 1:** The first order linear fitting model parameters of hardness in two stages (first stage:0–4 h; second stage:4–24 h).

Samples	a_1_	b_1_	R^2^	a_2_	b_2_	R^2^
DWS	1.71b	1.58 ab	0.92	8.48b	0.19a	0.85
DWS-0.5%MP	1.49a	2.60c	0.94	12.45c	0.13a	0.92
DWS-1%MP	1.81b	2.77d	0.98	12.98c	0.15a	0.98
DWS-3%MP	1.55a	1.62b	0.89	7.94 ab	0.18a	0.99
DWS-5%MP	1.55a	1.42a	0.86	7.67a	0.11a	0.95

Values in the same column having same letter are not significantly different (*p< 0.05*).

**Table 2:** Power law parameters for storage modulus (G′) of starch gels with different storage time.

Sample	2 h				4 h		
	K	z	R^2^		K	z	R^2^
DWS	156.97 ± 8.14c	0.143 ± 0.003c	0.986	DWS	242.26 ± 4.88c	0.071 ± 0.003a	0.974
DWS-0.5%MP	212.60 ± 10.76d	0.079 ± 0.002a	0.975	DWS-0.5%MP	269.45 ± 8.49d	0.080 ± 0.001b	0.995
DWS-1%MP	242.26 ± 13.88e	0.070 ± 0.002a	0.974	DWS-1%MP	322.26 ± 10.76e	0.077 ± 0.002 ab	0.991
DWS-3%MP	102.53 ± 6.40b	0.177 ± 0.016d	0.864	DWS-3%MP	223.98 ± 3.46a	0.084 ± 0.005b	0.943
DWS-5%MP	91.31 ± 3.32a	0.099 ± 0.002b	0.988	DWS-5%MP	230.10 ± 4.44 ab	0.070 ± 0.001a	0.993
	8 h				24 h		
	K	z	R^2^		K	z	R^2^
DWS	318.28 ± 4.63c	0.078 ± 0.004c	0.958	DWS	397.22 ± 8.47c	0.050 ± 0.003a	0.947
DWS-0.5%MP	386.19 ± 18.37d	0.082 ± 0.003c	0.981	DWS-0.5%MP	458.17 ± 5.38d	0.079 ± 0.002b	0.986
DWS-1%MP	455.66 ± 7.99e	0.049 ± 0.001a	0.980	DWS-1%MP	500.53 ± 15.30e	0.079 ± 0.001b	0.989
DWS-3%MP	295.06 ± 5.64b	0.063 ± 0.002b	0.988	DWS-3%MP	360.77 ± 4.65b	0.078 ± 0.001b	0.995
DWS-5%MP	280.06 ± 1.91a	0.098 ± 0.005d	0.954	DWS-5%MP	348.93 ± 5.28a	0.082 ± 0.003b	0.980

All results are expressed as the mean ± SD of three independent determinations. Values in the same column with different letters are significantly different (*p* ≤ 0.05). k and z = power law parameters, R^2^ = regression coefficient.

**Fig. 3:**
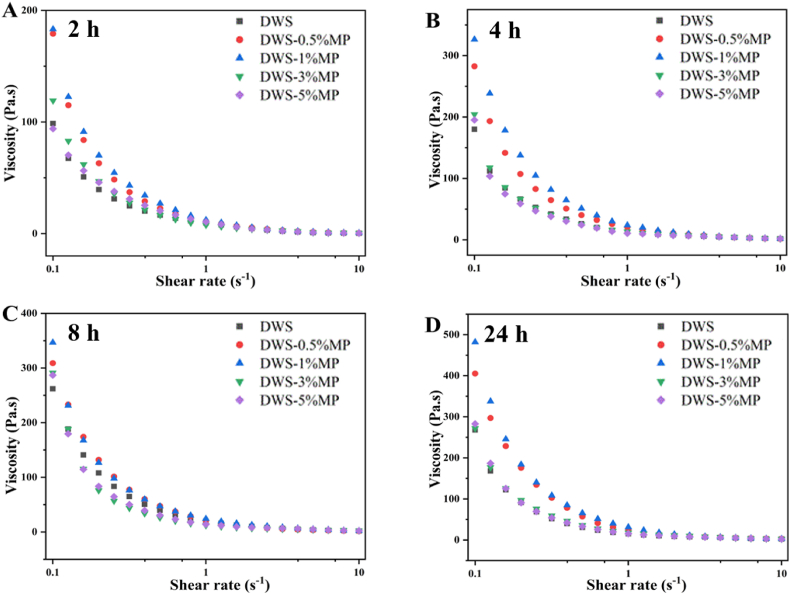
Apparent viscosity of DWS-MP gels at different storage times.

Apparent viscosity (steady rheological properties) is affected by particle shape, particle size, additive and interaction between particles (Agi et al., 2019). Effects of MP and retrogradation time on the apparent viscosity of DWS are shown in Fig. 3. Apparent viscosity of all starch gels decreased sharply with increasing shear rate, showing the characteristic (shear-thinning) of pseudoplastic fluid (Agi et al., 2019). In addition, with the increase of shear rate, the apparent viscosity decreased to a stable value. This might be due to the fact that starch molecules were oriented under the action of shear force (Chen et al., 2021). Regardless of the retrogradation time, DWS-0.5%MP and DWS-1%MP exhibited higher apparent viscosity values compared with pure DWS gel, indicating that adding low levels of MP (0.5 and 1%) promoted starch chains entanglement. However, the interaction between DWS and higher concentrations of MP was weaker. This was consistent with the variation of gel hardness. Furthermore, the apparent viscosity of DWS-MP gels was found to consistently increased as the increasing of retrogradation time. As the increase of storage time, starch molecules were aggregated and rearranged by intermolecular hydrogen bonds to form insoluble aggregates (gel network) (Fig. 1) (Liu et al., 2020), causing an increase in apparent viscosity.

### 3.3 Particle size distribution

**Fig. 4:**
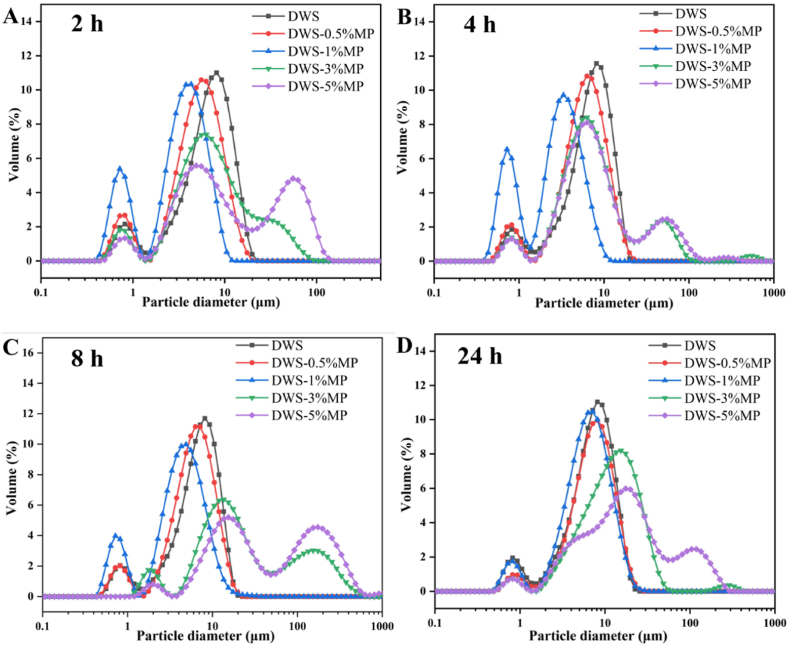
Size distribution of DWS-MP gels with storage time of (A) 2 h, (B) 4 h, (C) 8 h, and (D) 24 h.

The particle size distributions and parameters of DWS with or without MP at different retrogradation stages are shown in Fig. 4 and Table 3. The values of D_10_, D_50_ and D_90_ indicated that 10%, 50% and 90% of particles in the total starch particles were smaller than corresponding diameter, respectively (Chen et al., 2021). For the MP concentrations ranging from 0.5 to 1%, the mean particle size D_(3,2)_ of DWS after stored at 4 °C for 2 h decreased from 3.81 to 1.94 μm with increasing MP-concentration. With further increasing the amount of MP from 1 to 5%, however, the D_(3,2)_ values increased dramatically and reached to 4.77 μm. These results suggested that low concentrations of MP promoted the formation of DWS with a smaller size, whereas high concentrations of MP caused a larger particles size of DWS. The similar trend was also observed in the particle size parameters D_10_, D_50_, and D_90_.

**Table 3:** Particle size distribution, DO values (1047 cm^−1^/1022 cm^−1^), and relative crystallinity (RC) of DWS-MP gels with different retrogradation time.

2 h	D _(3,2)_	D_10_ (μm)	D_50_ (μm)	D_90_ (μm)
DWS	3.81 ± 0.24c	1.89 ± 0.24c	6.80 ± 0.50c	12.53 ± 1.06b
DWS-0.5%MP	3.13 ± 0.26b	1.09 ± 0.52b	5.20 ± 0.46b	10.12 ± 0.98 ab
DWS-1%MP	1.94 ± 0.13a	0.71 ± 0.02a	3.43 ± 0.26a	6.65 ± 0.57a
DWS-3%MP	4.13 ± 0.41c	2.37 ± 0.25cd	6.87 ± 0.72c	30.11 ± 3.65c
DWS-5%MP	4.77 ± 0.16c	2.64 ± 0.12d	7.17 ± 0.22c	44.32 ± 2.73d
4 h				
DWS	4.13 ± 0.09c	2.19 ± 0.06b	7.11 ± 0.17c	12.70 ± 0.25a
DWS-0.5%MP	3.66 ± 0.27b	2.24 ± 0.22b	5.94 ± 0.45b	11.50 ± 0.95a
DWS-1%MP	2.29 ± 0.16a	0.78 ± 0.03a	4.01 ± 0.31a	7.62 ± 0.69a
DWS-3%MP	4.69 ± 0.29d	2.70 ± 0.14c	6.84 ± 0.41c	42.70 ± 8.13b
DWS-5%MP	4.89 ± 0.31d	2.77 ± 0.15c	7.12 ± 0.44c	51.60 ± 8.82b
8 h				
DWS	4.53 ± 0.20c	2.47 ± 0.13b	7.83 ± 0.42c	14.10 ± 1.07a
DWS-0.5%MP	3.77 ± 0.32b	2.37 ± 0.23b	6.05 ± 0.53b	11.30 ± 1.18a
DWS-1%MP	2.44 ± 0.18a	0.81 ± 0.03a	4.24 ± 0.34a	8.68 ± 0.95a
DWS-3%MP	4.52 ± 0.32c	2.43 ± 0.18b	7.74 ± 0.63c	79.80 ± 18.07b
DWS-5%MP	7.42 ± 0.32d	3.45 ± 0.16c	15.80 ± 1.31d	112.00 ± 9.45c
24 h				
DWS	4.56 ± 0.92b	2.24 ± 0.20a	7.41 ± 0.53b	13.90 ± 1.12a
DWS-0.5%MP	4.42 ± 0.32b	2.87 ± 0.09b	6.53 ± 0.27a	11.13 ± 0.55a
DWS-1%MP	3.95 ± 0.16a	2.49 ± 0.11a	6.12 ± 0.27a	12.13 ± 0.29a
DWS-3%MP	7.26 ± 0.33c	3.93 ± 0.17d	12.33 ± 0.40c	29.00 ± 3.03b
DWS-5%MP	7.17 ± 0.21c	3.60 ± 0.12c	13.27 ± 0.21d	50.20 ± 6.58c

Means with different letters are significantly different in their respective column (*p* < 0.05).

During the retrogradation process, the debranched starch mainly undergoes three stages: (1) the starch chains are cross-linked to form a double helix cluster structure; (2) the semi-crystalline units are rearranged into nano-scale particles; (3) the particles grow and gather into aggregates (Cai and Shi, 2014; Wang et al., 2017). It has been reported that MP could promote the short/long-term retrogradation of maize starch (Luo et al., 2020). The changes of mean particle size may be due to that MP could efficiently accelerate the formation of granule structure of debranched starch molecules when low MP concentrations were added, which led to forming smaller granules. Adding high MP concentrations, however, might cause the self-aggregation between MP molecules, which would block DWS molecular crosslinking to form a smaller size granule structure (Xiao et al., 2021). Therefore, the values of D_(3,2)_ increased first and then decreased with the increasing of MP concentration. On the other hand, the values of those parameters of all DWS-MP gels increased with storage time, which suggested that the particles grew and gathered into aggregations during the retrogradation process.

### 3.4 Short-range ordered structure of starch

**Fig. 5:**
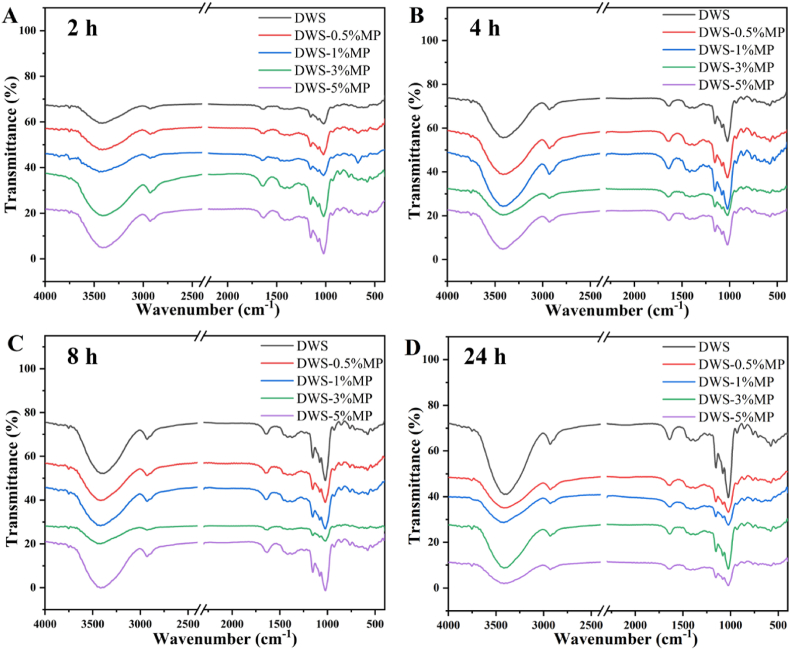
FT-IR spectra of DWS-MP gels DWS-MP gels with different storage time.

**Fig. 6:**
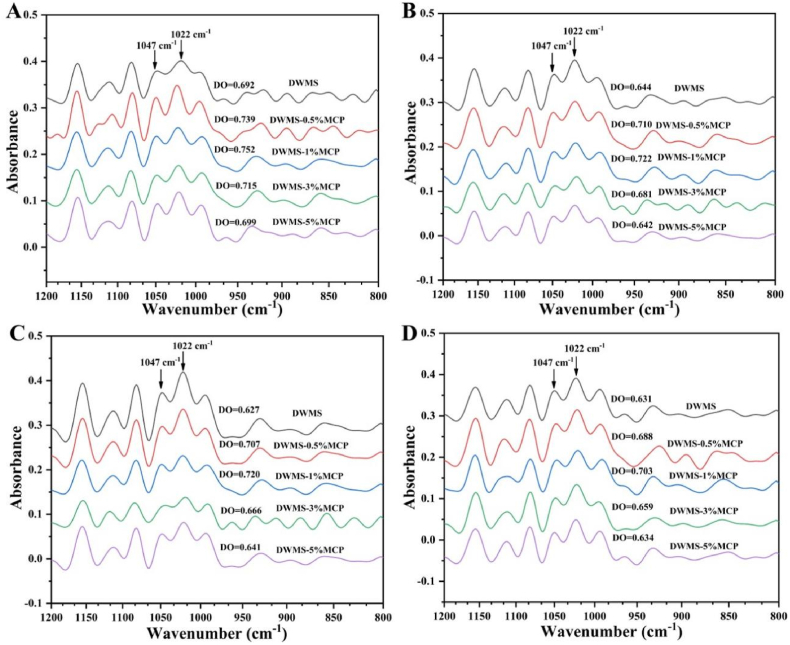
The deconvoluted FTIR spectra in the region of 800–1200 cm^−1^ of freeze dried DWS-MP gels with different retrograding time. (A) 2 h, (B) 4 h, (C) 8 h, and (D) 24 h.

The FT-IR spectra are used to analyze short-range ordered structure of starch gels (Sevenou et al., 2002). DWS gels treated with different MP concentrations and stored at 4 °C for 2, 4, 8, and 24 h were studied. The spectra curves of DWS treated with MP at different stored time were similar to untreated starch (Fig. 5), indicating that MP did not alter the chemical structure of starch. This was consistent with previous study in MP and sweet potato starch system (Ren et al., 2020). The deconvoluted spectra of all DWS-MP gels in the range of 800–1200 cm^−1^ are presented in Fig. 6 and the degree of short-range ordered (DO) (1047 cm^−1^/1022 cm^−1^) are showed in Table 3. The DO values increased first and then decreased with MP was added and DWS-1%MP exhibited the highest values of 0.752 compared to other groups after 2 h retrogradation, which indicated DWS-1%MP had a more ordered structure. This was due to the fact that MP promoted the cross-linking and rearrangement of starch molecules to form a more ordered network structure (Ren et al., 2020). However, only the appropriate addition of MP could significantly increase the ordered degree of DWS (Xiao et al., 2021).

It is worth noting that the DO values decreased significantly in the early step of retrogradation (<4 h), whereas decreased slightly in the later retrogradation stages (4–24 h) (Table 3). This result was contrary to previous reports that the DO of waxy maize starch-MP hybrid gels increased with the retrograding days (Luo et al., 2020). It is well known that the short-term regeneration is mainly determined by amylose (Zeng et al., 2022). These results might be attribute to the fact that the debranched starch almost consisted of linear short amylose, which retrograded very fast and subsequently the aggregation of granules occurred leading to a decrease in DO values. It has been proved in the test of particle size analyze that particle parameters increased clearly with stored time.

### 3.5 X-ray diffraction (XRD)

**Fig. 7:**
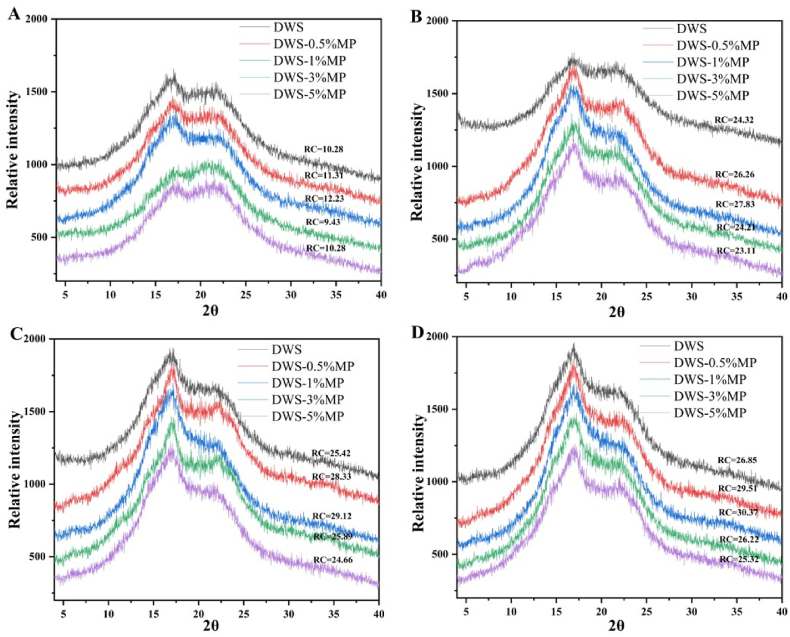
Wide angle X-ray diffraction spectra of freeze dried of freeze dried DWS-MP gels with different retrograding time.

The long-range order structure is associated to the stacking of the double helices in starch granules (Chen et al., 2019). The XRD spectra and relative crystallinity (RC) values of DWS-MP gels are showed in Fig. 7. Two diffraction peaks appeared at 17 and 22° of all DWS-MP gels after cooling for 2 h, although the peaks were very broad and not sharp (Fig. 7A). It is a typical B-type XRD pattern of starch and it has been reported that debranched starch with low solids concentration (5%) was prone to form B-typical crystalline structures (Cai and Shi, 2010). DWS-0.1%MP possessed the highest RC values, which was consistent with our previous study (Xiao et al., 2022). It was attributed to the crystallization of amylose (debranched starch) and promoting effect of appropriate MP during retrogradation. As cooling time increased (Fig. 7B), interestingly, it was obviously evidenced that diffraction peaks at 17° significantly increased and became sharp and RC values was enhanced (Table 3), which was consistent with previous studies (Wang et al., 2021; Yu et al., 2020). This was due to the debranched starch molecules rearrangement into crystalline areas at 4 °C environment. This rapid increase in RC period (0–4 h) might be related to the increase in gel hardness of DWS-MP gels.

However, the RC value of DWS-MP gels was almost no significantly difference between 8 h and 24 h retrogradation. It was possible that the rapid recrystallization and rearrangement of amylose was nearly completed after 4 h retrogradation, so that the subsequent cooling stored has little effect on the RC. In addition, since the particle size parameters in Table 3 increased significantly with storage time, suggesting that it was more likely to experience aggregation of starch granules. These results provided a good basis for understanding the structural changes in the retrogradation process of debranched starch.

### 3.6 Microstructure changes in the DWS-MP gels

**Fig. 8:**
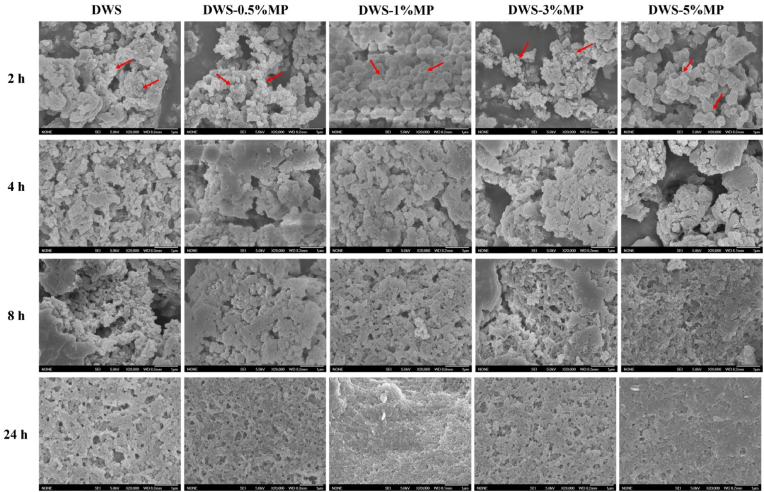
Scanning electron microscopy observation of DWS-MP gels with different retrogradation time.

The micrographs of DWS-MP gels stored at 4 °C for different time are shown in Fig. 8. It can be observed that all DWS gels with or without MP showed an irregular spherical structure (red arrow) and a clear aggregation phenomenon was found in all samples after 2 h retrogradation (Fig. 8). This was similar to previous studies that debranched starch displayed a high level of aggregation (Duyen and Hung, 2021; Qin et al., 2020). Compared with pure DWS gels, the starch particles exhibited a more homogeneous and smooth structure when low concentrations of MP were added, whereas the opposite effect occurred when high concentration of MP was added. This was in accordance with the particle size results. It was clarified that the nucleation and the subsequent growth of crystals could significantly affect the morphology and size of starch granules. At the start of the retrogradation, DWS molecules underwent rapid nucleation to form nanoscale nuclei. As recrystallization times increased from 4 to 24 h, however, the nucleation phases gradually shift to undertaking the aggregation of particles. Therefore, the size and the degree of aggregation increased with the extension of storage time. These results further indicated that retrogradation could significantly change the structure and morphology of DWS.

**Fig. 9:**
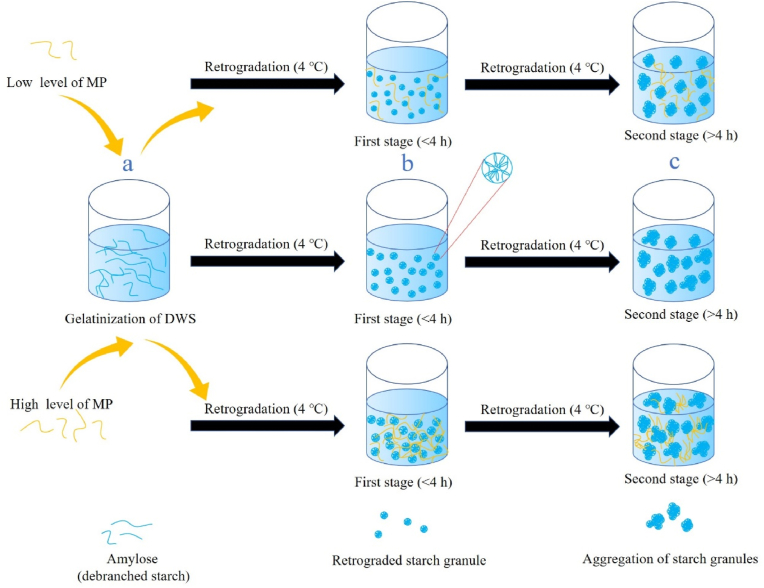
A schematic diagram depicting the potential mechanism of the retrogradation process for DWS.

To better understand the short-term retrogradation process of DWS, a schematic diagram depicting the potential mechanism is shown in Fig. 9. After heating, the initial DWS solution was clear and transparent (Fig. 1a) and consisted almost of amylose (debranched starch). During the cool storage at 4 °C, amylose reassociated into microcrystalline structure by double helix at a very fast rate, and then rearranged into spherical particles in the first stage (<4 h) (Fig. 9b). Gel hardness, particle size, and apparent viscosity increased significantly during this period, and the solution shifted from transparent to glossy, white opaque emulsion. After the first stage of retrogradation, since almost all amylose molecules were already involved in the formation of retrograded starch granules, the nucleation and cross-linking process almost ceased. In the next stage (>4 h), these starch particles mainly underwent the process of granule aggregation and adhesion to each other (Fig. 9c), leading to the formation of larger starch granules as demonstrated in SEM and particle size results. During the DWS retrogradation process, the addition of low level of MP induced the aggregation of DWS in a smaller space, forming smaller starch spheres. Nonetheless, the addition of high level of MP was more prone to the self-aggregation process of MP molecules, resulting in a less uniform system and the production of larger granules (DWS and MP).

## 4 Conclusions

In summary, only appropriate addition of MP can effectively promote the short-term retrogradation of DWS. The short-term retrogradation (1 day) of DWS could be divided into two phases. In the first phase (0–4 h), the gel hardness of DWS increased rapidly (b_1_ » b_2_), and a low concentration of MP can increase the rate of gelation. However, in the second phase (4–24 h), the gel hardness of DWS increased slowly (b_2_ < 0.2), and it was clearly demonstrated from SEM results and particles size test that this stage seemed to be dominated by the aggregation of starch granules. FT-IR results showed that the degree of short-range ordered (DO) monotonously decreased with storage time. Additionally, the results of XRD proved that relative crystallinity (RC) of DWS gel increased with storage time, and it increased significantly in the first phase while slight increase in the later stage. These results will enhance further understanding about the retrogradation process of debranched starch.

## CRediT authorship contribution statement

**Wenhao Xiao:** Writing – original draft, Visualization, Data curation. **Jinwang Li:** Writing – review & editing. **Mingyue Shen:** Writing – review & editing. **Qiang Yu:** Writing – review & editing. **Yi Chen:** Writing – review & editing. **Jianhua Xie:** Conceptualization, Software, Resources, Writing – review & editing, Supervision.

## Declaration of competing interest

The authors declare that they have no known competing financial interests or personal relationships that could have appeared to influence the work reported in this paper.
